# Rationale and Design of the PRegnancy and Infant DEvelopment (PRIDE) Study

**DOI:** 10.1111/ppe.12023

**Published:** 2012-12-05

**Authors:** Marleen M H J van Gelder, Reini W Bretveld, Jolt Roukema, Morac Steenhoek, Joris Drongelen, Marc E A Spaanderman, Dick van Rumpt, Gerhard A Zielhuis, Chris M Verhaak, Nel Roeleveld

**Affiliations:** a Department of Epidemiology, Biostatistics and HTA, Radboud University Nijmegen Medical CentreNijmegen, The Netherlands; b Department of Paediatric Pulmonology, Radboud University Nijmegen Medical CentreNijmegen, The Netherlands; c Department of Obstetrics & Gynaecology, Radboud University Nijmegen Medical CentreNijmegen, The Netherlands; d Department of and Medical Psychology, Radboud University Nijmegen Medical CentreNijmegen, The Netherlands; e Midwifery Practice CyclusNijmegen, The Netherlands; f Department of Cancer Registry and Research, Comprehensive Cancer Center the NetherlandsUtrecht, The Netherlands; g Department of Obstetrics and Gynecology, University Hospital MaastrichtMaastricht, The Netherlands; h SHO Centers for Medical DiagnosticsVelp, The Netherlands

**Keywords:** PRIDE Study, life-course, epidemiology, children, attention-deficit/hyperactivity disorder, pregnancy, asthma, autism

## Abstract

**Background:**

To optimise the health of pregnant women and their children by evidence-based primary and secondary prevention, more scientific knowledge is needed. To overcome the methodological limitations of many studies on pregnancy and child health, which often use a retrospective design, we established the PRIDE (PRegnancy and Infant DEvelopment) Study.

**Methods and Results:**

The PRIDE Study is a large prospective cohort study that aims at including 150 000–200 000 women in early pregnancy to study a broad range of research questions pertaining to pregnancy complications, maternal and child health, and adverse developmental effects in offspring. Women are invited to participate by their prenatal care provider before or at their first prenatal care visit and are asked to fill out web-based questionnaires in gestational weeks 8–10, 17, and 34, as well as biannually throughout childhood. In addition, a food frequency questionnaire and a paternal questionnaire are administered and medical records are consulted. Multiple validation studies will be conducted and paper-and-pencil questionnaires are available for women who cannot or do not want to participate through the Internet. For subgroups of participants, blood and saliva samples for genetic and biochemical analyses are being collected. The pilot phase, which started in July 2011, showed a response rate of 47%. Recruitment will eventually cover all of the Netherlands.

**Conclusions:**

We expect that this study, which will be the largest birth cohort in the world so far, will provide new insights in the aetiology of disorders and diseases that originate in pregnancy. The PRIDE Study is open for collaboration.

Exposures that occur during gestation and early childhood may be associated with diseases and disorders that manifest themselves at birth, during childhood, or even later in life. Indeed, various associations between prenatal or early-life exposures and diseases that are typically diagnosed in childhood have been reported. For instance, maternal use of acetaminophen during pregnancy, *in utero* exposure to maternal smoking, and delivery by caesarean section have all been implicated to play a role in the aetiology of childhood asthma.^1^–^3^ Furthermore, increased rates of attention-deficit/hyperactivity disorder (ADHD) have been found after prenatal exposure to labetalol, preterm birth, and organophosphate exposure,^4^–^6^ while gestational diabetes, vaginal bleeding, and neonatal jaundice may increase the risk of autism.^7^,^8^ Numerous associations between early-life exposures and diseases that occur in adolescence or adulthood have been reported as well. Classical examples include associations between birthweight and the occurrence of ischemic heart disease,^9^,^10^ obesity,^11^ and diabetes.^12^,^13^ However, the results of many of these studies focusing on early-life exposures and later diseases are inconsistent. Most likely, many risk factors for disorders such as birth defects, respiratory conditions, autism, ADHD, and childhood cancer, are as yet unknown. Identifying possible risk factors for these and other disorders is a crucial step in the development of preventive measures.

For methodological reasons, birth cohort studies are recommended to answer these types of research questions. By following subjects over time, plausible and potentially causal explanations for the observed associations may be addressed and temporal changes in various factors, such as maternal mental health and blood pressure, may be monitored. As early as the 1950s, the value of birth cohort studies was acknowledged and two large studies, the California Child Health and Development Studies and the Collaborative Perinatal Project,^14^,^15^ started enrolment at the end of that decade. To date, a total of 17 true longitudinal birth cohorts with at least 5000 participants have been described which all enrolled women prospectively during pregnancy (Table 1).^14^–^33^ Two of these included 100 000 women, and three others are planning to do so. The reported response rates ranged from 30% to as high as 96% with a median response rate of 75%. Regarding data collection, a variety of methods have been used (Table 2). Only six studies collected self-reported data in all three trimesters of pregnancy. Most studies attempted to follow-up their cohorts into childhood. Biological samples were mostly obtained from subgroups of participants only. In addition, almost all birth cohort studies consulted medical or obstetric records to obtain clinical data and linkages to medical registries were often established. In 10 cohorts, mothers or infants were medically examined as well.

**Table 1:** Overview of longitudinal birth cohort studies with ≥5000 participants which enrolled women prospectively during pregnancy

Cohort	Location	Enrolment period	Timing of enrolment	Sample size	Reported response rate
Aarhus Birth Cohort^16^,^17^	Aarhus, Denmark	Sept. 1989–	<16 weeks of gestation	>20 000	75%
ABCD Study^18^	Amsterdam, the Netherlands	Jan. 2003–March 2004	First prenatal care visit	8266	67%
ALSPAC^19^,^20^	Avon, England	April 1991–Dec. 1992 (EDD)	Majority in early pregnancy	14 541	85%
Born in Bradford^21^	Bradford, England	March 2007–2010	Gestational weeks 26–28	10 000	NR
C-ABCS^22^	Anhui, China	Nov. 2008–Oct. 2010	55% first trimester; 45% second trimester	16 766	94%
CCHDS^14^	California, USA	1959–1966	Early pregnancy	20 754	NR
CPP^15^	USA	1959	First prenatal care visit	60 000	NR
Danish National Birth Cohort^23^,^24^	Denmark	1996–2003	First prenatal care visit	100 000	30%
Generation R^25^,^26^	Rotterdam, the Netherlands	April 2002–Jan. 2006	76% early pregnancy; 21% mid-pregnancy; 3% late pregnancy	8880	61%
HHf2^27^	Odense and Aalborg, Denmark	April 1984–April 1987	Gestational week 36	11 980	87%
Hokkaido Study^28^	Hokkaido, Japan	Feb. 2003–	<13 weeks of gestation	20 000	NR
JECS^a^	Japan	2011–	Early pregnancy	100 000	NR
National Children's Study^29^,^30^	USA	2009–	First trimester	100 000	NR
NINFEA^a^	Italy	2005–	Pregnancy	10 000	NR
Northern Finland Birth Cohort^31^	Oulu and Lapland, Finland	1966 (EDD)	Gestational weeks 24–28	12 058	96%
Norwegian Mother and Child Cohort Study^32^,^33^	Norway	1999–2007	Gestational weeks 17–18	100 000	44%
UK Birth Cohort Study^a^	UK	2013–	NR	110 000	–

a Peer-reviewed cohort profile is not available, information was extracted from the study websites in July 2012 (JECS: http://www.env.go.jp/en/chemi/hs/jecs/; NINFEA: https://www.progettoninfea.it/index_en; UK Birth Cohort Study: http://www.esrc.ac.uk/funding-and-guidance/tools-and-resources/research-resources/surveys/bcf.aspx).

ABCD, Amsterdam Born Children and their Development; ALSPAC, Avon Longitudinal Study of Parents and Children; C-ABCS, China-Anhui birth cohort study; CCHDS, California Child Health and Development Studies; CPP, Collaborative Perinatal Project; EDD, estimated date of delivery; HHf2, Healthy Habits for Two; JECS, Japan Environment and Children's Study; NR, not reported.

**Table 2:** Methods of data collection used in existing longitudinal birth cohort studies

	Self-reported data			Biological samples		
			
Cohort	Method	Timing prenatal	Post-partum until	Mother	Infant	Other data sources
Aarhus Birth Cohort	Q	Trimester 1	–	–	–	Medical records, registries
ABCD Study	Q	Early pregnancy	Adulthood	Blood	–	Medical records, registries, physical examinations
ALSPAC	Q	Multiple times	Adulthood	Blood, urine, placenta, hair, toe nail	Cord blood, umbilical cord, blood, urine, saliva	Medical records, environmental monitoring, home observations, education records, physical examinations
Born in Bradford	I	Trimester 3	–	Blood, urine	Cord blood	Medical records, registries, physical examinations
C-ABCS	Q	Multiple times	15 years^a^	Blood	Blood	Medical records, physical examinations
CCHDS	I	Multiple times	Adolescence	Blood, placenta	–	Medical records, registries, physical examinations
CPP	I	Multiple times	–	Blood	–	Medical records, observations, physical examinations
Danish National Birth Cohort	I	Multiple times	18 months	Blood	Umbilical cord	Registries
Generation R	Q	Multiple times	Adulthood	Blood, urine	Cord blood	Medical records, physical examinations
HHf2	Q	Trimester 3	–	–	–	Medical records
Hokkaido Study	Q	Trimester 1	School age	Blood, hair, breast milk	Cord blood	Medical records
JECS	Q + I	Early pregnancy	13 years	Blood, urine, hair, breast milk	Cord blood, blood, urine, hair	Medical records, environmental monitoring
National Children's Study	I	Multiple times	21 years	Blood, urine, placenta, breast milk, saliva, hair, vaginal swabs	Cord blood, umbilical cord, meconium, blood, urine, saliva, hair	Medical records, environmental monitoring, physical examinations
NINFEA	Q	Pregnancy	18 months	Saliva	–	Registries
Northern Finland Birth Cohort	Q	Weeks 24–28	14 years	–	–	Medical records, registries, physical examinations
Norwegian Mother and Child Cohort Study	Q	Multiple times	7 years	Blood, urine	–	Registries
UK Birth Cohort Study	Q	NR	7 years	Not specified	–	Medical records, registries, environmental monitoring, physical examinations

a Only in subgroup.

ABCD, Amsterdam Born Children and their Development; ALSPAC, Avon Longitudinal Study of Parents and Children; C-ABCS, China-Anhui birth cohort study; CCHDS, California Child Health and Development Studies; CPP, Collaborative Perinatal Project; HHf2, Healthy Habits for Two; I, interview; JECS, Japan Environment and Children's Study; NR, not reported; Q, questionnaire.

The existing birth cohort studies provide sufficient data to test a wide range of hypotheses, which already resulted in many research papers. For example, by November 2011 over 500 research papers were published from the Avon Longitudinal Study of Parents and Children, 230+ papers from the Danish National Birth Cohort, and more than 750 publications from the Collaborative Perinatal Project.^34^ However, the existing birth cohorts also generate new hypotheses and subsequently pose new research questions which cannot be answered with the data collected, such as possible health risks associated with cellphone use and the effects of organic food consumption by either women or children. In addition, common behaviours may change over time and may affect maternal, fetal, or infant health of this and future generations.^35^ To overcome the methodological problems associated with retrospective study designs and to test hypotheses that cannot be studied in the existing birth cohort studies because of power limitations or lack of sufficiently detailed data, we established a new prospective birth cohort study, the PRegnancy and Infant DEvelopment (PRIDE) Study.

## The Dutch prenatal care system

The PRIDE Study is based in the Dutch prenatal care system that is unique in the Western world, although it inspired changes in the prenatal care systems of Canada, New Zealand, and the UK. In the Netherlands, midwives are autonomous medical practitioners that are qualified to provide full prenatal care to all women with uncomplicated pregnancies and deliveries. The first prenatal care visit usually takes place around gestational week 8 and frequent contacts are scheduled throughout pregnancy. In case of risk factors or complications, women are referred to a secondary or tertiary care midwife or gynaecologist. In 2008, 84% of pregnant women started their prenatal care in a primary care setting. Approximately half of the pregnant women (47%) started labour in primary care, 33% of women delivered under supervision of a primary care midwife, and the home birth rate was almost 25%.^36^

## Goals of the PRIDE Study

We aim to include 150 000–200 000 Dutch women in early pregnancy in the PRIDE Study to evaluate a broad range of research questions pertaining to maternal and child health and adverse developmental effects in offspring. The primary objective of the PRIDE Study is to identify factors to which women may be exposed during pregnancy that potentially affect the health of the future mother or her unborn child at any point in life. The secondary aim of the PRIDE Study is to evaluate specific aspects of preconceptional, prenatal and perinatal care in the Netherlands (e.g. expectations, efficiency, and cost-effectiveness of counselling, screening, and prenatal diagnostic procedures and treatment options). In light of these objectives, a multitude of research questions have been formulated for the PRIDE Study. The priority exposures and outcomes are listed in Table 3.

**Table 3:** Priority exposures and outcomes that guide the data collection of the PRIDE Study

Priority exposures	Priority outcomes
Preconception care	Pregnancy complications
Maternal genotype	Late miscarriage
Maternal anthropometrics and blood pressure	Preterm birth
Medication use, including vaccines	Low birthweight and macrosomia
Maternal chronic conditions and illnesses	Birth defects
Maternal depression and depressive symptoms	Apgar score
Maternal physical and emotional stress	Developmental delays
Environmental endocrine disruptors	Wheezing, asthma, and other respiratory conditions
Occupational exposures	Autism
Nutrition and vitamin supplements	Attention-deficit/hyperactivity disorder (ADHD)
Life style habits, including smoking, alcohol consumption, and cellphone use	Infectious diseases in childhood
Housing conditions and home environment	Obesity (maternal and infant)
Social determinants	Diabetes (maternal and infant)
Breast feeding	Hypertension (maternal)

## Study design

### Recruitment

The health care providers in prenatal care play a central role in the enrolment of pregnant women into the PRIDE Study. They are contacted through the professional organisations of midwives, gynaecologists, and general practitioners. Participating health care providers give verbal and written information about the PRIDE Study to pregnant women and encourage them to visit the PRIDE Study website (http://www.pridestudy.nl). On this website, women can fill out the study questionnaires using a personal login code provided by their health care provider. Basically all Dutch pregnant women are eligible for participation; the only two exclusion criteria are (1) maternal age <18 years (in 2008, only 0.1% of deliveries occurred among Dutch women in this age group^36^), and (2) >16 weeks pregnant at intake. Women are asked to participate on a completely voluntary basis and give informed consent digitally through the Internet. In 2011, 99% of Dutch women aged 25–45 years had access to the Internet, 91% through a broadband connection.^37^ Paper-and-pencil consent forms and questionnaires are available for women who cannot or do not want to participate through the Internet. In addition, every effort is made to improve response rates, including the careful use of design elements in the study questionnaires,^38^ participation in monthly raffles, and regular newsletters. Reassuringly, the Danish National Birth Cohort and the Norwegian Mother and Child Cohort Study did not find indications for considerable bias in exposure–outcome associations resulting from non-participation.^24^,^33^ The PRIDE Study has been approved by the Regional Committee on Research involving Human Subjects. Recruitment started in the first region in July 2011 and will gradually be expanded to encompass all of the Netherlands in 2012. Inclusion is expected to be finished by the end of 2015.

### Data collection

**Figure 1:**
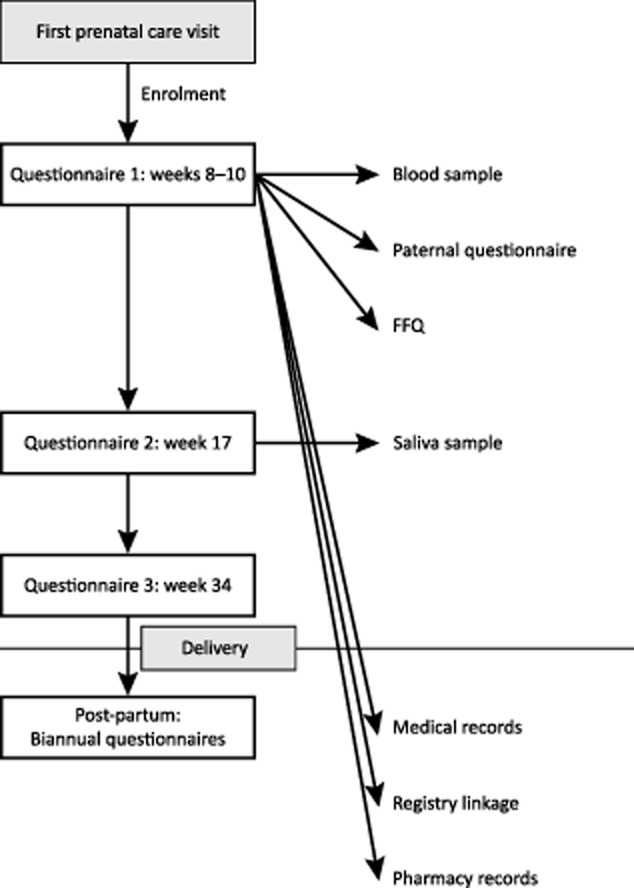
Structure of the data collection for the PRIDE Study. The solid boxes represent the primary mode of data collection; the dashed boxes indicate the additional components. FFQ, Food Frequency Questionnaire.

The complete structure of the data collection for the PRIDE Study is shown in Figure 1. In principle, pregnant women are invited to participate in the study by their midwife, gynaecologist, or general practitioner through email and/or a regular letter just before or at their first prenatal care visit. They are asked to complete web-based questionnaires as early in pregnancy as possible, usually during gestational weeks 8–10 (aetiologically relevant period for birth defects), in weeks 17 (before 20-week ultrasound) and 34 (just before delivery for most pregnancies), as well as biannually after giving birth until the infants reach the age of 21 years, starting with the first postnatal questionnaires 2 and 6 months after the expected date of delivery. Specific questionnaires are available in case of a miscarriage or preterm birth. In an extensive review of the literature, it was determined that this relatively new method of data collection is suitable for use among women of reproductive age.^39^ Based on the results of this review, we incorporated some important measures in the data collection phase of the PRIDE Study to enhance data quality, such as the use of a mixed-mode design (web-based and paper-and-pencil questionnaires) and initiation of various validation studies for data on, for instance, medication use and pregnancy complications. The questionnaires were especially designed for the PRIDE Study using expertise and example questionnaires from previous research and the existing prospective birth cohort studies. In collaboration with researchers from various medical specialties, including obstetrics, midwifery, paediatrics, psychiatry, psychology and physiology, existing paper questionnaires or parts thereof were selected, modified, and tailored to our web-based application. Whenever possible, validated questionnaires and methods were used and incorporated into the PRIDE Study, such as the Edinburgh Depression Scale (EDS), the Hospital Anxiety and Depression scale (HADS), and the Ages and Stages Questionnaires. For exposures for which this was not feasible, including medication use and occupational exposures, an intricate web-based system was developed, in which a tree-like structure with multiple choice questions fed by underlying databases of possible exposures guides the participants to the appropriate answer. In addition to the standard prenatal and postnatal maternal questionnaires, the women may fill out a detailed food frequency questionnaire around gestational weeks 8–10 and invite the future biological father to complete a questionnaire focusing on exposures in the 3 months before the index pregnancy.

In the informed consent form, permission is asked to consult pharmacy and medical records during pregnancy and after birth. If permission is granted, we will also obtain data from the Netherlands Perinatal Registry, the National Institute for Public Health and the Environment, and the Comprehensive Cancer Center the Netherlands to verify specific exposures reported in the questionnaire, to collect more detailed information on both exposures and outcomes, and to assess the potential for bias resulting from non-participation.

Subgroups of participants, including those reporting a diagnosis of certain chronic conditions (e.g. depression, chronic hypertension) or use of selected drugs, such as antidepressants, antihypertensive medications, and statins, as well as all participants living in predefined geographic areas, are being invited to donate four 4.5 mL blood samples in the first part of pregnancy for genetic and biochemical analyses. These regions, which are located throughout the country in both rural and urban areas, are defined by the service areas of the blood draw facilities of the Centers for Medical Diagnostics, at which non-fasting blood samples are taken during routine blood sampling among pregnant women or by special invitation. From three of the four blood samples, serum and plasma is being separated and subdivided into eight units (four serum, three plasma, and one erythrocytes); the fourth sample is whole blood for DNA extraction. All blood samples are stored at −80°C until laboratory analyses. To increase the numbers for future genetic analyses, women who are not included in blood sampling may later be asked to provide a saliva sample using DNA self-collection kits send by regular mail.

In the second prenatal questionnaire, women are also asked to donate a saliva sample to measure cortisol levels, which can be measured from saliva in a reliable and stress-free way^40^ and reflect the biologically active (unbound) fraction of serum cortisol.^41^ The participating women are sent a polypropylene numbered tube (Salivette® Cortisol, Sarstedt AG & Co, Nümbrecht, Germany) by regular mail and are asked to collect a saliva sample within 10 min after waking up on a weekday, while not brushing their teeth, eat, drink or smoke before taking the sample. The samples are sent back by mail and subsequently stored at −20°C for biochemical analyses in nested case–control designs. Although collecting multiple samples across several days is preferable to assess diurnal changes in cortisol and intra-individual variability, this is not feasible in the PRIDE Study because of participant burden and costs. Salivary cortisol levels in samples collected immediately after waking up have shown a higher day-to-day correlation than afternoon samples,^42^ and they will be used as a marker for maternal stress, which is one of the priority exposure of the PRIDE Study.

### Power calculation

The PRIDE Study is not only designed for research on relatively rare exposures and outcomes, but also to study interactions between exposures including gene–environment interactions and the risks of more common diseases, such as asthma, autism, and ADHD. Although the PRIDE Study will initially include 150 000–200 000 pregnant women and permission for linkage with medical records and registries as well as exposure to many factors is assessed in the first prenatal questionnaire, we will not be able to conduct analyses using data from all subjects because of refusals, loss to follow-up, and missing values. Therefore, the power calculations are based on a conservative estimate of the size of our study population, namely 120 000, but if more subjects can be included, study power will increase. In the Netherlands, 177 713 infants were born in 2008, of which 7.7% were born preterm (<37 weeks of gestation), 6.2% had a low birthweight (<2500 g), and 2.8% had a major birth defect.^36^ Therefore, the expected numbers of cases in our study population of 120 000 children are 9240, 7440 and 3360 for preterm birth, low birthweight, and major birth defects, respectively. However, the number of infants with specific birth defects will be much lower, which limits the number of associations that can be studied with sufficient study power for these outcomes. Prevalences of diseases that manifest in childhood are less readily available for the Netherlands, but may be estimated using prescription rates for drugs used in the treatment of these diseases. Anti-asthma and ADHD medication have been prescribed to 4.9% and 2.1% of children, respectively,^43^,^44^ corresponding to at least 5880 and 2520 children with asthma and ADHD in our study population. Using these figures, we calculated the minimal exposure prevalences needed to demonstrate a relative risk of at least 2.0 with a type I error of 5% and a type II error of 20%. The results of these calculations are shown in Table 4, which indicates that it will be possible to reliably study even rare exposures and combinations of exposures in relation to the development of various outcomes within the PRIDE Study.

**Table 4:** Minimal exposure prevalences needed to demonstrate a relative risk of ≥2.0 (α = 0.05, study power 80%), based on 120 000 children

Outcome	Prevalence outcome (%)	Expected no. of cases	Minimal exposure prevalence (%)
Preterm birth	7.7	9240	0.08
Low birthweight	6.2	7440	0.10
Major birth defect	2.8	3360	0.23
Asthma	4.9	5880	0.13
ADHD	2.1	2520	0.31

ADHD, attention-deficit/hyperactivity disorder.

### Results of the pilot phase

In the pilot phase (July 2011–April 2012), seven out of eight invited midwife practices and one university hospital enrolled pregnant women into the PRIDE Study. A total of 976 eligible women were invited, of which 459 (47.0%) agreed to participate. This response rate is slightly higher than those in similar birth cohort studies in Denmark (30%) and Norway (44%).^23^,^24^,^32^,^33^ The completion rate of the first questionnaire was >95% at a median gestational age of 10 weeks. Less than 1% of the participants preferred paper-and-pencil questionnaires. The consent rates for the additional data collection components were high: 88.9% for the paternal questionnaire (response rate among men 41.2%), 81.9% for the food frequency questionnaire, 88.2% for linkage with the Netherlands Perinatal Registry, 74.7% for review of medical records, 80.2% for review of pharmacy records, and 76.5% for saliva sampling. Blood samples of 266 women were stored. No major problems in the enrolment or data collection methods were identified, but based on feedback from the participants and careful evaluation some minor adjustments were made in content and length of the questionnaires and in technical and logistical aspects of the data collection.

### Perspectives

From a public health perspective, it is of major importance to determine whether exposures that occur early in life are causally related to diseases and disorders that manifest themselves at birth, during childhood, or later in life. This scientific information may contribute to the implementation of evidence-based preventive measures for a large number of disorders, including birth defects, low birthweight, asthma, autism, ADHD, obesity, cardiovascular diseases, and diabetes. However, establishing a causal relation between intrauterine or childhood exposures and diseases that occur later in life is challenging, in particular as exposure to the factor of interest and to confounding factors may have taken place years or even decades before the outcome occurs.^45^ Prospective birth cohort studies with large sample sizes may overcome many of the methodological shortcomings of cross-sectional and retrospective studies in perinatal and paediatric epidemiology.

With a total of 150 000–200 000 pregnancies, the PRIDE Study will be the largest longitudinal birth cohort study conducted so far. Its prospective design in combination with the use of web-based questionnaires allows us to measure a broad range of exposures in detail in aetiologically relevant time frames. In addition, biomonitoring can be used to validate part of the self-reported data, while linkage with medical records and existing registries enables us to reliably collect clinical data on the health of participating mothers and children. This approach strongly increases data quality by minimising the chance of information bias, especially when compared with retrospective study designs. We believe that this study will provide new and useful insights about the potential role of many prenatal and early-life exposures, such as medical drug use during pregnancy, fever and infections, maternal stress, pregnancy complications, diet, genetic factors, and parental occupational exposures, in the aetiology of a large number of diseases. In the end, these insights may be used to improve maternal and child health by developing and implementing preventive measures in prenatal care and during childhood.

The PRIDE Study is open for collaboration with external groups. As recruitment and data collection are still ongoing, additional measurements may be implemented in the study if warranted. Requests for collaboration and proposals for projects should be sent to project@pridestudy.nl. Requests and proposals are discussed in the PRIDE Study Data Sharing Committee with respect to their study aims, feasibility, overlap with ongoing studies, and financial contributions. After approval by this committee and the Regional Committee on Research involving Human Subjects, a contract including mutual obligations (e.g. data delivery, publication plan, and contribution to the PRIDE Study infrastructure) will be drawn up and collaboration can commence.
